# Designing optimizing procedures for task switching to ensure efficiency in the hospital laboratory

**DOI:** 10.1038/s41598-021-92116-z

**Published:** 2021-06-16

**Authors:** Young-Jin Kim, Garam Kim, Sangil Kim, Dawoon Jung, Minwoo Park

**Affiliations:** 1 Finance Fishery Manufacture Industrial Mathematics Center On Big Data, Pusan National University, Busan, 46241 Korea; 2 Institut Für Mathematik, Technische Universität Berlin, Berlin, Germany; 3 Department of Mathematics, Pusan National University, Busan, 46241 Korea; 4 Department of Laboratory Medicine, ST. Vincent’s Hospital, The Catholic University of Korea, Suwon, Korea

**Keywords:** Health care, Mathematics and computing

## Abstract

This study aims to improve the efficiency of task switching in hospital laboratories. In a laboratory, several medical technicians perform multiple tasks. Technicians are not aware of the marginal amount of time it takes to switch between tasks, and this accumulation of lost minutes can cause the technician to worry more about the remaining working time than work quality. They rush through their remaining tasks, thereby rendering their work less efficient. For time optimization, we identified work changeover times to help maintain the work quality in the laboratory while reducing the number of task switching instances. We used the turnaround time (TAT) compliance rate of emergency room samples as an indicator to evaluate laboratory performance and the number of task switching instances as an index of the task performer perspective (TPP). We experimented with a monitoring system that populates the time for sample classification according to the optimal time for task switching. Through the proposed methodology, we successfully reduced not only the instances of task switching by 10% but also the TAT non-compliance rate from 4.97 to 2.66%. Consequently, the introduction of new methodology has greatly increased work efficiency.

## Introduction

Business process management (BPM) has been introduced to efficiently manage business processes in all industries. Elizabeth et al. suggested that "BPM is a systematic, structured approach to analyze, improve, control, and manage processes to improve the quality of products and services"^1^. This approach to process research can be referred to by several terms such as "process simplification", "process improvement”^2^, and "reengineering"^3^.

Thus, research for improving the quality of products and services is actively conducted in the industrial field, and in many cases, the goal is to improve customer satisfaction. For this reason, the execution of more complicated business processes is required, and often workers are then required to perform an excessive amount of work.

BPM has been investigated in various ways and from different perspectives. Lee et al. conducted a study on how the entire process of BPM was executed by considering the workload of the participants^4^. In this case, the addition of new personnel and allocation of business functions was managed through BPM. Eder et al. suggested that personal schedules allow us to (1) analyze the workload of a participant and (2) support the scheduling of activities to reduce turnaround times as well as the number of violations of temporal constraints^5^. Zhao and Stohr suggested a methodology for turnaround time prediction, time allocation, and task prioritization. In their method, the expected execution time is allocated to each unit task^6^.

With the emergence and advancements in the field of data science, data analysis has become an effective tool for BPM. The application of data science in BPM is referred to as process mining or automated business process discovery (ABPD)^7^. Process mining aims to improve the understanding and efficiency of the process through data analysis of the event log. It is based on a combination of model-based approaches and data mining^8,9^. It can analyze work efficiency and provide optimal guidance through its operations^10,11^. Through process mining, a mathematical optimization problem was constructed to reduce the workload of actual participants and maintain work efficiency.

In this study, we assumed that one person would take charge of the business processes and execute multiple tasks. The processing order of each sample task was fixed and had a time limit. The processing method for all the samples was the same, whereas the time limit varied with the circumstance through which the samples were processed. When multiple samples were mixed, the worker was required to switch tasks during the process within a short time. It is challenging for a worker with little experience to make a heuristic decision when switching tasks. Similar to the law of supply and demand, increasing the number of task switching instances enhances the intensity of work and reduces the number of samples beyond the time limit. Conversely, reducing the number of task switching instances lowers the intensity of the tasks but increases the number of samples beyond the time limit.

Rogers and Monsell utilized an alternating runs procedure to point out the alternation cost, in which subjects performed the first task several times, then the next, and so forth^12^. The procedure compared the performance between the pure task block (AAAA) and an alternating task block (AABB), when A and B were a single task. This was later confirmed by Fagot, who added an alternating ABAB block, and then it was redrawn by Pashler^13,14^. The results showed that AAAA had the lowest cost, followed by AABB and ABAB. Using the hospital example, we apply the following values:

- : Pre-treatment process of $i$ th sample with a time limit of 60 min.
- : Analytical process of the $i$ th sample with a time limit of 60 min.

If the samples are handled in $P_{1} P_{2} P_{3} P_{4}$ order, then $A_{1} A_{2} A_{3} A_{4}$ is the best order to run the next set of processes. Subsequently, the entire sequence becomes $P_{1} P_{2} P_{3} P_{4}$–$A_{1} A_{2} A_{3} A_{4}$, which implies that task switching occurs once. However, in reality, it might be $P_{1}$–$A_{1}$–$P_{2} P_{3}$–$A_{2} A_{3}$–$P_{4}$–$A_{4}$, when considering the time limit of the sample processing. To clarify, task switching is likely to occur five times within the process $P_{1}$–$A_{1}$–$P_{2} P_{3}$–$A_{2} A_{3}$–$P_{4}$–$A_{4}$. However, when there is sufficient time to wait between the first $P_{1}$ and the second $P_{2}$, it can be processed as $P_{1} P_{2} P_{3}$–$A_{1} A_{2} A_{3}$–$P_{4}$–$A_{4}$ or –$P_{1} P_{2} P_{3} P_{4} A_{1} A_{2} A_{3} A_{4}$. It is evident that the latter is the best option since the task switching action is much lower than in the other scenarios.

The purpose of this study was to maintain or improve the results of sample processing. By modeling the outcome of sample processing during a short time limit and task transitions such as the supply and demand curve, we found the optimal time to wait for the first $P_{1}$ and reduced the amount of task switching. Specifically, this implies that the number of transitions is reduced by properly allocating tasks to the person in charge, rather than distributing tasks or task switching, which generally refers to multitasking situations presented sequentially without time overlap^15–17^.

This study focuses on improving the turnaround time (TAT) for clinical chemistry tests in an emergency room (ER) situation in which the tasks to be handled are piled up and task switching actions must be chosen by the operator himself. Thus, to reduce the number of task switching instances, the operator selects the timing of the switching based on the information expressed in the provided algorithm to increase task efficiency; given that the working capabilities of each person in charge are the same.

Note that these problems are prevalent in hospital laboratories. ER samples must be processed before the outpatient and inpatient samples. Although the sample processing process is the same, ER samples with a short time are preferentially treated. The time spent processing each sample is referred to as the turnaround time or TAT. Notably, laboratory TAT is one of the most important indicators of the level of laboratory service and is used by several clinicians to determine the quality of a laboratory^18^.

In “Background and methods” section, we give detailed descriptions of the task switching problem and present a proposed mathematical optimization method by defining variables as well as the problem statement. In addition, we delineate the algorithm for finding work intervals. In “Results” section, we explain the results of the algorithm, which suggests the optimal work interval for sample processing, with several graphs. Moreover, we demonstrate how the suggested optimal interval reduces task switching instances for medical technicians and compare task switching data from 2018 and 2019. In “Discussion” section, we conclude with a summary of comments for researchers and provide future scope that can be used to enhance efficiency in hospitals.

## Background and methods

### Background and data

**Figure 1:**
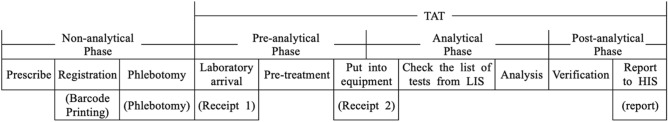
Laboratory workflow in a ward at the St. Vincent’s hospital.

Figure 1 illustrates the procedure for performing clinical chemistry tests in a ward at the St. Vincent's hospital. The procedure can be explained as follows: When a physician prescribes an examination, a nurse prints the barcodes, collects blood from the patient, and then records the blood collection time in the Laboratory Information System (LIS). Then, the samples are transported to the laboratory through an auto-track or pneumatic tube. Once the samples arrive at the laboratory, the time of Receipt 1 is recorded in the LIS by a medical technologist. Even if Receipt 1 is omitted, it is still possible to proceed to the next step. Next, the samples undergo a pre-treatment process (centrifugation, removing caps of samples, and mounting samples in a rack). Subsequently, when the pre-processed samples are put into the equipment, Receipt 2 is recorded in LIS, and examinations are performed. After the analysis, the results are sent by the LIS. The results are then verified by a medical technologist and reported to the Hospital Information System (HIS) automatically through the LIS.

In this study, we restricted the TAT of intra-laboratory activities. TAT was classified into three phases based on the following three time points: ‘Receipt 1’, ‘Receipt 2’, and ‘report’. The ‘Barcode Printing’ and ‘Phlebotomy’ tasks were classified in a non-analytical phase, and the goal was to have an ER TAT of one hour or less (based on the TAT guideline of St. Vincent’s Hospital).

It is important to note that there are different definitions of TAT. Lundberg described the ’total testing cycle’ as a series of nine steps: ordering, collection, identification, transportation, preparation, analysis, reporting, interpretation, and action^19,20^. Laboratory TAT is determined by the period of three phases of testing: pre-analytical (order to preparation), analytical (analysis), and post-analytical (report to action)^21–23^. Furthermore, TAT is sometimes used to describe the interval between when a test is requested, and when a treatment decision is made^24–27^. However, many laboratories restrict their definition of TAT to intra-laboratory activities, based on the argument that factors other than TAT are noncontrollable and cause a non-analytical delay^28^.

Steindel and Howanitz suggested that reasonable components of TATs are 15 min for both the order of collection and collection-to-receipt times and 30 min for the receipt-to-verification time for urgent samples from an ER. Another method suggested by the National Academy of Clinical Biochemistry required the collection-to-reporting TAT to be one hour or less^29^. Our study focuses on improving the overall TAT for clinical chemistry tests conducted in an ER.

**Figure 2:**
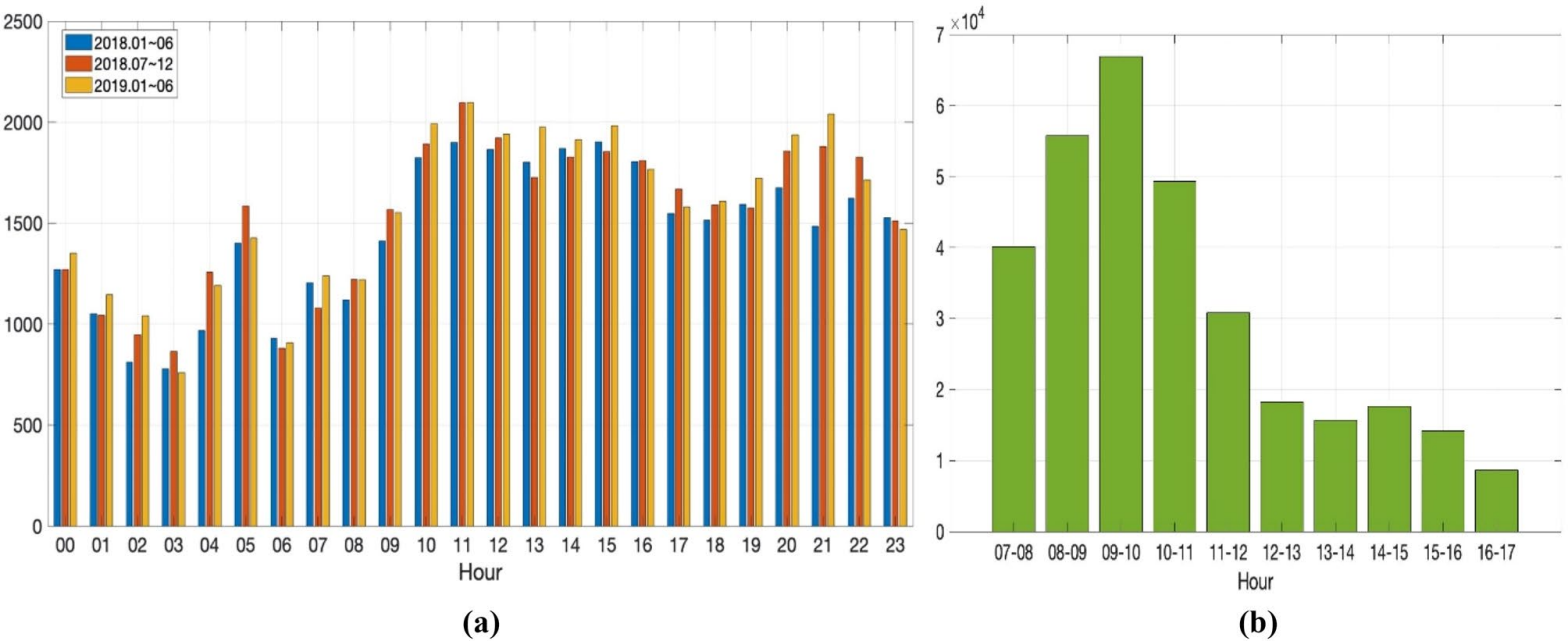
(**a**) Number of ER samples arriving at the department of laboratory medicine by time from January 2018 and to July 2019. (**b**) Number of outpatient samples at the St. Vincent’s hospital in 2019.

Figure 2a shows the number of ER samples that arrive at the department of laboratory medicine in a day. It can be seen that the workload varies significantly by the time zone. Further, during the daytime, the overall sample numbers increase, in addition to the absolute workload for outpatient and inpatient examinations and ER samples. In practice, the number of outpatient and inpatient samples is much greater compared to that of the ER samples seen in Fig. 2b.

We considered the number of workers based on the time of the day and work transition time. The number of workers staffed per shift was as follows: during the daytime (07:00–17:00), there were four workers; in the evening (17:00–22:00), there were three workers, and at night (22:00–07:00), there were two workers. Notably, there were more workers during the daytime as compared to the other times of the day, to ensure that the workload is evenly distributed. Therefore, counting the number of instances of task switching during the daytime was not considered important.

As the workload increases, task switching occurs more frequently, and the number of excessively delayed TAT samples also increases. The workload can increase directly or by the effect of various events. Some medical technicians may perform excessive task switching when the workload increases. We observed the tendency to work with as many samples as possible through real work on centrifugation at the pre-analytical phase. We consider that it is a natural phenomenon to try to minimize task switching as much as possible. However, it is challenging for a technician to determine the optimal timing for each moment. Therefore, we conclude that it is crucial to use an objective method rather than a heuristic one to find the optimal performance interval that complies with the TAT.

To determine the optimal timing, we applied mathematical optimization (described in “Mathematical optimization for finding the optimal unit work interval” section below) to the dataset provided by St. Vincent’s hospital. The data shown in Fig. 1 as well as the blood collection time, pre-reception time, reception time, and final report time for the ER biochemical samples were collected from January 2018 to December 2019 by researchers through the HIS and approved by the IRB Ethics Committee. All methods were carried out in accordance with relevant guidelines and regulations of ST. Vincent’s Hospital. All experimental protocols were approved by the Institutional Review Board (IRB) of ST. Vincent’s Hospital. The requirement for informed consent was exempted from the IRB of ST. Vincent’s Hospital due to its retrospective nature.

### Mathematical optimization for finding the optimal unit work interval

Mathematical optimization involves the selection of the best element (with regard to certain criterion) from a set of available alternatives. The standard form of an optimization problem is as follows:

$$ \begin{aligned}    & Minimize~\quad f\left( x \right) \\     & subject\;to\quad g\left( x \right) \ge 0~ \\  \end{aligned} $$

where $f\left( x \right)$ is the objective function to be minimized and $g\left( x \right)$ is the constraint ^30^. Multi-objective optimization is an integral part of optimization activity and is of tremendous practical importance, since almost all real-world optimization problems are ideally suited to be modeled using multiple conflicting objectives^31^. The generalized form of multi-objective optimization can be defined as follows:

$$ \begin{aligned}   Minimize\quad  &  & F\left( x \right) = \left\{ {f_{1} \left( x \right),~f_{2} \left( x \right),~ \ldots ,~f_{m} \left( x \right)} \right\} \\    subject\;to\quad  & g\left( x \right) \ge 0 \\     & x_{j}  \ge 0\quad ~j = 1,~2, \ldots ,n \\  \end{aligned} $$

where $x$ is the vector of variables, $~f_{i} \left( x \right)$ is the $i$th objective function, and $g\left( x \right)$ is the constraint. This involvement of multiple considerations can cause a dilemma: Which of these optimal solutions must one choose? To address this question, the effort must be focused on finding a set of trade-off optimal solutions by considering all objectives to be important. In such a case, Deb Kalyanmoy suggests the principle for an ideal multi-objective optimization procedure: Find multiple optimal trade-off solutions and choose one that includes a higher-level of information^31^. Hence, we look for the best possible trade-off solutions to our optimization problem and choose the solution with the highest level of importance.

For clarity, let us consider an example of a flower delivery service. We would need to determine the optimal delivery distance from our office, which would require the least amount of fuel consumption and low labor costs. We can define this scenario through a mathematical optimization problem:

$$ \begin{aligned}   find & \quad \mathop {{\text{argmin}}}\limits_{x} ~~\left\{ {f_{1} \left( x \right),~f_{2} \left( x \right)} \right\} \\     & subject\;to\quad x \ge 0~ \\  \end{aligned} $$

where $x$ is the distance, the first objective function $~f_{1} \left( x \right)$ is the fuel consumption, the second objective function $~f_{2} \left( x \right)$ is labor cost. The distance should be a positive value, so $x \ge 0$ is the constraint and $\mathop {{\text{argmin}}}\limits_{x} h\left( x \right)$ are the points for which $h\left( x \right)$ has its smallest value. Once multiple trade-off distances are found, we choose one optimal distance taking into account the factors we mentioned earlier.

Our goal is to minimize task switching, which requires us to reduce the number of tasks. To address the aforementioned problems, the ER samples in 2018 were analyzed and modeled as a mathematical optimization problem. An optimal sample processing time interval was proposed based on the algorithm.

The original data $D = \{ A_{i}  = \left( {x_{i} ,{\text{~}}y_{i} ,{\text{~}}z_{i} } \right)|~i \in I~\}$ is the input where index $i$ is the order of the sample, $A_{i}  = \left( {x_{i} ,{\text{~}}y_{i} ,{\text{~}}z_{i} } \right)$ is the time data of the $i$th sample, which is a $1 \times 3$ row vector, and $x_{i}$, $y_{i}$, and $z_{i}$ are the respective times of Receipt 1, Receipt 2, and report to HIS for the $i$th sample. Then, for each duration time (min) $M$, the algorithm provided three outputs: modified data $D_{M}  = \{ \left( {x_{i} ,{\text{~}}y_{i} ,{\text{~}}z_{i} } \right)|~i \in I~\}$, the number of task switching instances $\left( {l_{M} } \right)$ and TAT satisfaction rates $\left( {r_{M} } \right)$. That is, let $M$ be the duration (min). For a fixed $k$, let $I_{k}  = {\{i~|}~y_{i}  - x_{k} ~ \le M,~{\text{for~all}}~i > k\}$, and for all $i \in I_{k} ~,~~y_{i}  = x_{k}  + M$. That is, for the fixed sample $k$, if $y_{i}$ is less than or equal to, $x_{k}  + M,$ then substitute it with $x_{k}  + M$. Before $M$ minutes have elapsed from $x_{k}$, make $y_{i}$ have the same time point of receipt 2 as $x_{k}  + M.$ Therefore, $y_{i}$, which has the same time point of Receipt 2, is regarded as a single task and is replaced by $y_{l} ~,~~l \in I$. Consequently, for any $M$, we want to find an optimal $M_{0}$ based on the task switching instances and TAT satisfaction rates.

**Figure 3:**
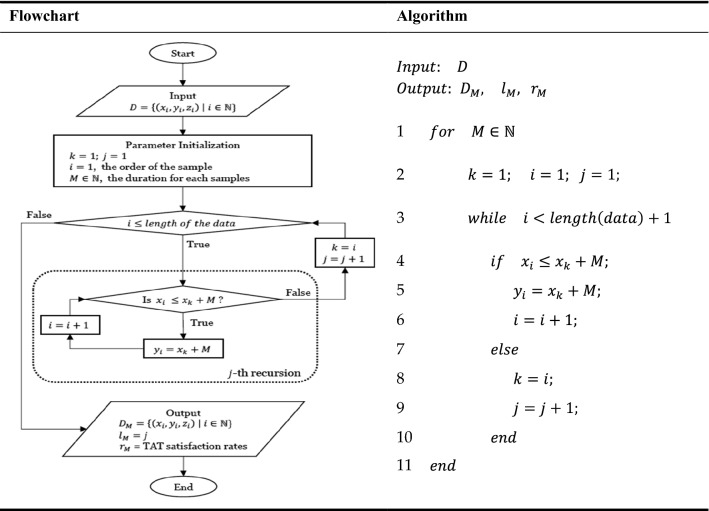
Flowchart and proposed algorithm for task switching.

In the following, we define the optimal problem of task switching in mathematical form, and provide a simple graphic representation in Fig. 3 to illustrate the algorithm for task switching.

$$ \begin{aligned}    & find\;\;M_{0}  = \mathop {{\text{argmin}}}\limits_{M} {\text{~~}}g\left( {f_{1} \left( M \right),{\text{~}}~f_{2} \left( M \right)} \right) \\     & where\;\;f_{1} \left( M \right) = 1 - r_{M} {\text{~}},{\text{~~}}f_{2} \left( M \right) = l_{M}  \\  \end{aligned} $$

## Results

### Algorithm and interpretations

Using the aforementioned algorithm, we find the optimal work interval to increase work efficiency and TAT satisfaction rates.

**Figure 4:**
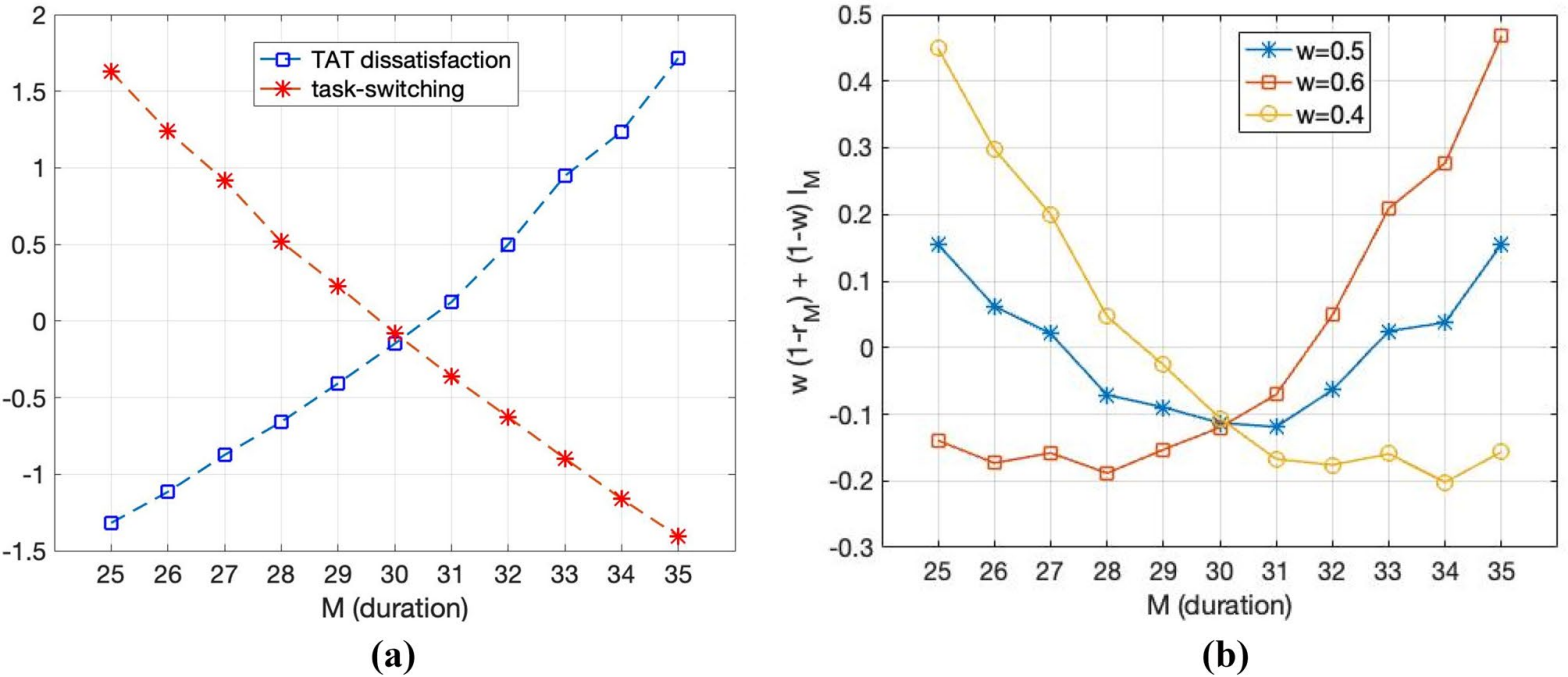
(**a**) Graph with normalized variables $1 - r_{M}$ and $l_{M}$. For each $M$ (duration), the red line shows the number of tasks and the blue line denotes the proportion of the samples that exceeded the TAT, 60 min. (**b**) Linear combination of task switching and TAT dissatisfaction reflecting different weights $\left( w \right)$.

Figure 4a shows the number of task switching instances $\left( {l_{M} } \right)$ and TAT dissatisfaction rates ($1 - r_{M}$) according to the change in $M$. It can be seen that the larger the $M$, the fewer the instances of task switching. It is linearly reduced, whereas the TAT dissatisfaction rate linearly increases. This situation is similar to the “Supply and Demand” curves. When $M$ is at 30 min, the normalized amount of task switching and normalized TAT dissatisfaction rates are met, which indicates that the optimization is performed using two variables.

Furthermore, we assign weights to both variables. Notably, it can be interpreted as the variable measured by the hospital. In Fig. 4a, the two variables are given equal weights, and in Fig. 4b, they are represented by blue lines with asterisk markers. This implies that TAT dissatisfaction and task switching are of the same level of importance.

Let us suppose that reducing the workload of the laboratory is more essential than satisfying TAT. Then, we give task switching and TAT dissatisfaction a weight of 0.6 and 0.4, respectively. To rephrase,$~0.4 \times \left( {1 - r_{M} } \right) + 0.6l_{M}$, which is represented by a yellow line with the circle marker in Fig. 4b. In this case, $M_{0}$ was 34 min. This means that the accumulated samples should be processed before 34 min has elapsed. Consequently, the laboratorian can perform other tasks for up to 34 min before the accumulated samples are placed into the equipment. Therefore, since switching from other tasks to Receipt 2 is less likely to occur, the workload of laboratorians has been curtailed, but TAT dissatisfaction has increased.

Let us next suppose that the hospital wants to increase the rate of TAT satisfaction. Hospitals might want laboratorians to work more frequently (less task switching) to improve the quality of medical services. In this case, weight was given to reduce the rate of TAT dissatisfaction. Then, let us give a weight of 0.6 and 0.4 to TAT dissatisfaction and task switching, respectively. This is represented by the red line with a square mark in Fig. 4b.

In this case, $M_{0}$ was 28 min. After a maximum of 28 min, the accumulated samples were placed into the equipment. By working more frequently, the rate of TAT satisfaction can be increased. As such, it can be seen that the optimal working interval is formed from 28 to 34 min, depending on which variable is more important. Hence, we suggest an interval of 30 min by assuming the general situation of the hospital.

### Improved result of laboratory performance

Changing tasks are commonly related to changes in valid stimulus–response mapping. Therefore, technicians should prepare and implement another task; commonly assumed to be performed by cognitive control^32–35^. Hence, a monitoring system was developed to alert technicians about switching their work based on the aforementioned algorithm. This system automatically informs the medical technician 30 min after the sample arrives at the laboratory, using the optimal work interval we discovered from the algorithm, so that they could meet the TAT guideline, which is 1 h or less in St. Vincent’s hospital. This monitoring system has been used since 2019, and data from January to August 2019 and January to August 2018 were used for the analysis.

**Figure 5:**
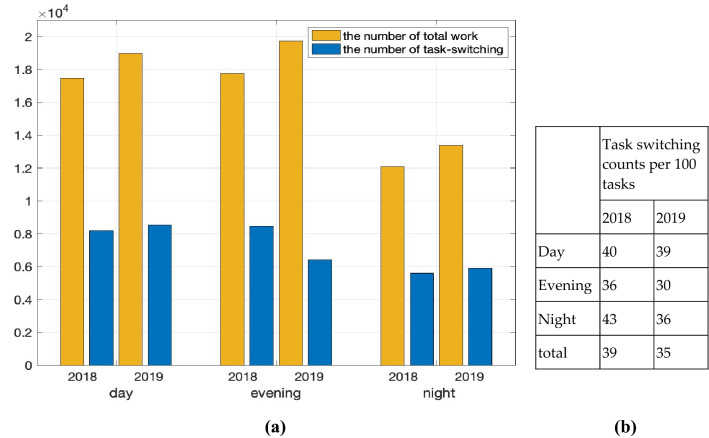
(**a**) Number of total works at the laboratory, and the number of task switching instances. (**b**) Task switching counts for every 100 works for each duty in the hospital.

Prior to analyzing the results, a technician's duty is divided into three parts: 7:00–15:00 is the daytime duty, 15:00–23:00 is the evening duty, and 23:00–7:00 on the following day is the night duty. Figure 5a shows the total number of tasks and the number of switched tasks before and after introducing the monitoring system in the laboratory. Each bar is divided by duty and year. This indicates that the number of total workloads was much higher in 2019 than in 2018. Apart from the amount of work increased (except day duty since most outpatients were visiting the hospital in the daytime), the task switching counts decreased year after year. Figure 5b compares the task switching counts for every 100 tasks for each duty. It shows that the number of task switching instances for all duties has decreased and that the number decreased the most during the night duty.

Considering that the total amount of tasks in 2019 from January to August was 50,000, the reduction of about 20,000 switched tasks allowed medical technicians to reduce the burden of work and increase their efficiency by 40%.

To reiterate, our goal was to reduce the number of task switching instances for workers to perform more efficiently by maintaining or improving turnaround time. We see from these results that the number of task switching instances decreases, but the increase in the TAT excess sample did not increase efficiency. Figure 5b shows the results of the processed samples in 2018 and 2019.

Table 1 shows a reduction in the proportion of samples exceeding TAT compared to the total samples received by the Department of Laboratory Medicine. There were improvements in the overall hours. In particular, as Hamilton-Fairley et al. claimed, tasks performed at night should be minimized to deliver safe patient care^36^. This was proven in that the effects of diminished task switching were the most outstanding during the night duty. Outlier management had a significant effect on night duty as well. Thus, a significant improvement was observed in both TAT and outlier management.

**Table 1:** Overall statistics of samples: mean and standard deviation (mean ± std) of each phase and overall TAT of clinical chemistry tests at ER, within 60 min and after 60 min.

		Sample no. (%)	Pre-analytical phase	Analytical + post-analytical Phase	Overall TAT
2018. 1–12	Overall ER sample	71,623 (100%)	20.26 ± 8.66	16.90 ± 8.66	37.16 ± 12.63
	Sample reported within 60 min	67,996 (94.94%)	19.39 ± 6.87	15.81 ± 5.18	35.20 ± 8.44
	Sample reported after 60 min	3627 (5.06%)	36.66 ± 17.61	37.34 ± 23.23	74.00 ± 19.64
2018. 1–6	Overall ER sample	34,875 (100.00%)	20.05 ± 8.51	16.93 ± 9.16	36.98 ± 12.82
	Sample reported within 60 min	33,141 (95.03%)	19.23 ± 6.77	15.79 ± 5.32	35.02 ± 8.43
	Sample reported after 60 min	1734 (4.97%)	35.58 ± 18.05	38.75 ± 25.41	74.33 ± 21.84
2019. 1–6	Overall ER sample	37,571 (100.00%)	19.13 ± 7.71	16.33 ± 6.90	35.46 ± 10.64
	Sample reported within 60 min	36,572 (97.34%)	18.67 ± 6.55	15.76 ± 5.08	34.43 ± 8.21
	Sample reported after 60 min	999 (2.66%)	35.90 ± 19.47	37.12 ± 20.03	73.02 ± 18.45

**Figure 6:**
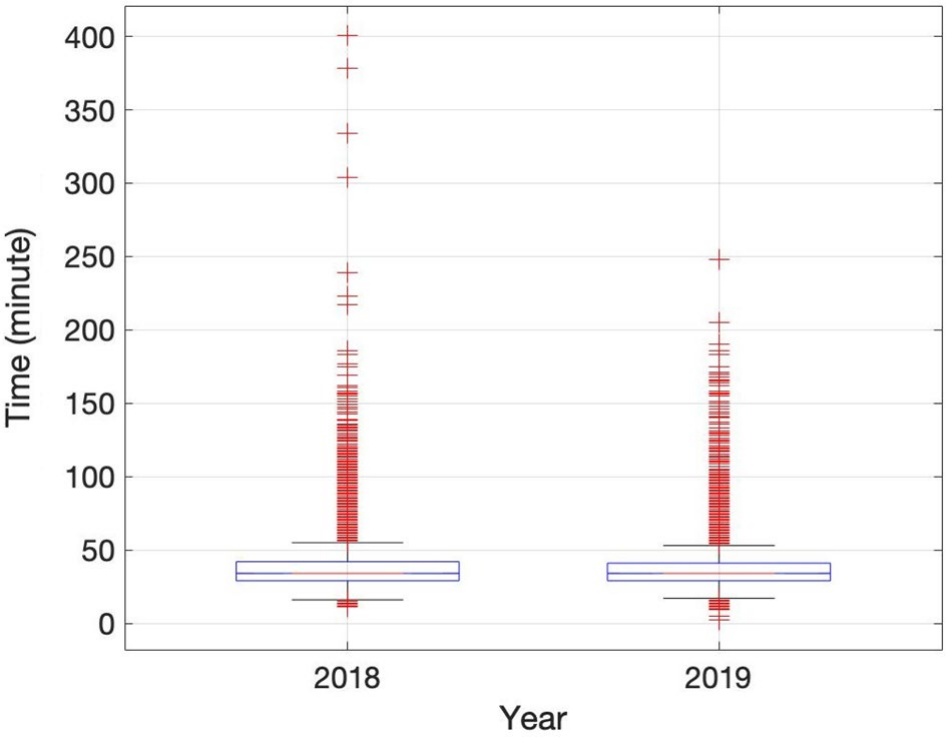
Boxplot with the TAT of the laboratory for each year. In each box, the central mark indicates the median, and the bottom and top edges of the box indicate the 25th and 75th percentiles, respectively. The outliers are plotted individually using the ‘+’ symbol.

In Fig. 6, the 25th percentile and median were 29 min and 34 min, respectively. The 75th percentile in 2018 and 2019 were 42 and 41 min, respectively. The number of samples that exceed the TAT guideline significantly decreased in 2019; from 2284 to 1465 samples. The ratio of samples exceeding the TAT guidelines improved significantly from 0.048 to 0.028.

We compared and analyzed the data from 2019 when the monitoring system was introduced with the 2018 data for the same time period. We defined task switching as the task change from Receipt 2 to another task in laboratory. It decreased from 39 to 35% of the total amount of work and shows a marked decline in the evening and night duties.

In addition, the number of samples that meet the TAT guidelines increased, as did the management of outliers, which we determined was 240 h. In addition, Fig. 6 shows that the maximum value of the extreme outlier was reduced, and the total number of outliers was also reduced. Therefore, we achieved satisfactory results by (1) improving the efficiency of the laboratory and the TAT satisfaction ratio and (2) reducing the work burden on medical technicians in the laboratory.

### Staff satisfaction survey

Since 2019, a monitoring program that enables technicians to be alerted to the optimal timing for task switching has been utilized. We carried out a survey about the program by including all eight technicians in the chemistry laboratory. In Table 2, the results show that all of them responded positively. In particular, all employees in the evening and night shifts responded strongly to questions 1 and 2.

**Table 2:** Results of the survey on the program's review.

	Strongly agree	Agree	Neutral	Disagree	Strongly disagree
1. Do you think the program helps the work process?	6	2	0	0	0
2. Do you think the program is effective in reducing TAT?	5	3	0	0	0
3. What did you find useful while using this program?	A total of eight answers were summarized as follows 1. It is good to be able to keep track of the current progress of clinical chemistry work while doing various tasks at the same time 2. Providing information about samples that was almost missed 3. Easy to know information about the current situation				

## Discussion

Currently, medical process management is gaining importance in hospitals owing to the strong competition for survival. Many activities are being carried out to improve the quality of medical services to increase customer satisfaction. In order to improve TAT, the work process workflow can be analyzed to make up for insufficient equipment and process it to be analyzed first^37^. Alternatively, the TAT performance can be improved by automating receipt or reporting^38,39^. It is also possible to improve tat by managing emergency tests by middleware linked to equipment^40^.

The laboratories at the St. Vincent’s Hospital were considered in this study since these hospitals are currently making efforts to improve their medical services through performance improvement activities. The hospital manages the temporal TATs and responds to the patients. The method presented in this study required many samples from the ER and inpatient areas. This made task completion highly efficient through the day; however, the number of test samples increased significantly for the outpatients. The results are extremely challenging during the day duty. There is, however, a need for additional research that considers outpatient and inpatient samples to increase the efficiency of the tasks performed during the daytime, especially during rush hours. Although we found the best TAT by analyzing the sample time taken over the past two years, we did not consider the fact that the optimal timing might differ depending on the amount of work and the type of duty as well as the change in the number of workers. Additionally, changes in the number of samples due to additional events were not considered.

Moreover, with the development of medical technology, the types of examinations required for suspected diseases may change, and the time required for each examination may vary. If the research that predicts the required time for each of these sample scenarios in real-time is supported, research that updates the reference points every hour can be conducted in consideration of the working environment of the laboratory. In essence, when we estimate the time required between different tasks according to the season, day of the week, and time zone, we can find the real time reference point considering the number of employees at that time.

As noted in the guidance of the World Health Organization (WHO) regional office for Europe on developing the quality and safety strategies using a health system, strengthening of healthcare quality reinforces a country’s health care system^41^. Moreover, WHO assesses that collecting and using data is an essential part of modern quality improvement. In this current era of the big data, a several studies for improving medical diagnosis and health care decision using artificial intelligence or machine learning have been conducted, although only a few studies focus on the medical quality of the ER^42–45^. Hence, in the future, we plan to develop an artificial intelligence-based method of updating the reference points every hour by considering the various events of a laboratory in real-time, rather than using a program for collectively determining the batch reference points used in this study. Ultimately, regardless of how optimized the program is, awareness and training on TAT are crucial in this regard. Therefore, we will continue our investigation in this direction in the future.

## Conclusion

This study presented a method for turnaround time (TAT) management by reducing the number of task switching instances in order to increase work efficiency in a chemistry laboratory without an additional workforce. The fact that medical technicians perform multiple tasks simultaneously and various events occur unexpectedly have a significant negative impact on the laboratory TAT. In addition, the distinct features of each sample during the analysis and frequent changes in workload in real-time make it challenging for workers to find the optimal time to adjust their workload.

With regard to aspect $I_{k}$, the reference time was set to accommodate as many samples as possible for each step. Therefore, setting an appropriate reference time for each step is necessary. Otherwise, the sum of all the reference times from all the steps would exceed the desired TAT. In other words, it is necessary to find the task switching time marker that satisfies the desired TAT with the least amount of work by setting the appropriate task switching time based on the finite time and (human) resources. For this reason, we determined the optimal timing of task switching through mathematical optimization.

For the convenience of the workers, we developed a monitoring system to alert the technicians about when they should switch their tasks based on the aforementioned algorithm. The developed system automatically informs the medical technician at a specified time after the sample arrives at the laboratory. The system provides an optimal work interval derived using mathematical optimization equations. This system was introduced to the hospital laboratory to evaluate the efficiency of the quality of the lab. It showed that the ratio of TAT guideline compliance and the work efficiency of the medical technicians improved owing to the use of this system.
